# Modeling and Surveillance of Reporting Delays of Mosquitoes and Humans Infected With West Nile Virus and Associations With Accuracy of West Nile Virus Forecasts

**DOI:** 10.1001/jamanetworkopen.2019.3175

**Published:** 2019-04-26

**Authors:** Nicholas B. DeFelice, Ruthie Birger, Nathaniel DeFelice, Alexandra Gagner, Scott R. Campbell, Christopher Romano, Michael Santoriello, Jennifer Henke, Jeremy Wittie, Barbara Cole, Cameron Kaiser, Jeffrey Shaman

**Affiliations:** 1 Department of Environmental Medicine & Public Health, Icahn School of Medicine at Mount Sinai, New York, New York; 2 Earth Institute, Columbia University, New York, New York; 3 Department of Epidemiology of Microbial Diseases, Yale School of Public Health, Yale University, New Haven, Connecticut; 4 Department of Environmental Health Sciences, Mailman School of Public Health, Columbia University, New York, New York; 5 Division of Infectious Diseases, University of California, Davis School of Medicine, Sacramento; 6 Division of Pulmonary/Critical Care Medicine, University of California, Davis School of Medicine, Sacramento; 7 Communicable Disease Program, Chicago Department of Public Health, Chicago, Illinois; 8 Arthropod-Borne Disease Laboratory, Suffolk County, Department of Health Services, Yaphank, New York; 9 Coachella Valley Mosquito and Vector Control District, Indio, California; 10 Disease Control Branch, Riverside County, Department of Public Health, Riverside, California

## Abstract

**Importance:**

West Nile virus (WNV) is the leading cause of domestically acquired arboviral disease.

**Objective:**

To develop real-time WNV forecasts of infected mosquitoes and human cases.

**Design, Setting, and Participants:**

Real-time forecasts of WNV in 4 geographically dispersed locations in the United States were generated using a WNV model-inference forecasting system previously validated with retrospective data. Analysis was performed to evaluate how observational reporting delays of mosquito WNV assay results and human medical records were associated with real-time forecast accuracy.

**Exposures:**

Mosquitoes positive for WNV and human cases.

**Main Outcomes and Measures:**

Delays in reporting mosquito WNV assay results and human medical records and the association of these delays with real-time WNV forecast accuracy.

**Results:**

Substantial delays in data reporting exist for both infected mosquitoes and human WNV cases. For human cases, confirmed data (n = 37) lagged behind the onset of illness by a mean (SD) of 5.5 (2.3) weeks (range, 2-14 weeks). These human case reporting lags reduced mean forecast accuracy for the total number of human cases over the season in 110 simulated outbreaks for 2 forecasting systems by 26% and 14%, from 2 weeks before to 3 weeks after the predicted peak of infected mosquitoes. This period is the time span during which 47% of human cases are reported. Of 7064 mosquito pools, 500 (7%) tested positive; the reporting lag for these data associated with viral testing at a state laboratory was a mean (SD) of 6.6 (2.6) days (range, 4-11 days). This reporting lag was associated with decreased mean forecast accuracy for the 3 mosquito infection indicators, timing, magnitude, and season, by approximately 5% for both forecasting systems.

**Conclusions and Relevance:**

Delays in reporting human WNV disease and infected mosquito information are associated with difficulties in outbreak surveillance and decreased real-time forecast accuracy. Infected mosquito lags were short enough that skillful forecasts could still be generated for mosquito infection indicators, but the human WNV case lags were too great to support accurate forecasting in real time. Forecasting WNV is potentially an important evidence-based decision support tool for public health officials and mosquito abatement districts; however, to operationalize real-time forecasting, more resources are needed to reduce human case reporting lags between illness onset and case confirmation.

## Key Points

**Question:**

What are the operational challenges limiting effective implementation of a real-time West Nile virus (WNV) forecasting system?

**Findings:**

In this modeling study of historical and real-time mosquito WNV assay results and human medical records, delays in data reporting for both infected mosquitoes and human WNV cases were associated with a reduction in average WNV forecast accuracy.

**Meaning:**

For public health departments and mosquito abatement districts to integrate forecasting effectively into operational decision making, the relaying of real-time health and environmental surveillance data should be prioritized.

## Teaser

This modeling study examines delays in reporting of human and mosquito West Nile virus infection and the association between these delays and accurate real-time forecasting of West Nile virus outbreaks.

## Introduction

During the past 30 years, the Western Hemisphere witnessed the arrival of a number of unexpected and important arthropod-borne viruses (arboviruses): dengue trickled in with small outbreaks that became more aggressive in the 1990s,^1^ West Nile virus (WNV) emerged in 1999,^2^ chikungunya in 2013,^3^ and in 2015, Zika virus became a global health emergency.^4^ Arboviruses are viruses transmitted by arthropods, predominantly mosquitoes and ticks, and are often maintained in complex zoonotic transmission cycles.^5^ Arboviruses are normally maintained in wild animal reservoirs, such as nonhuman primates or birds, making elimination difficult and prediction of spillover to humans challenging.^6,7^ As a consequence, effective allocation of public health resources is complicated and often reactive. Quantitative decision support tools that accurately and rapidly forecast the progression of arbovirus outbreaks and future human case numbers have the potential to help public health officials better prevent, identify, and treat these emerging infectious diseases.

In the continental United States, the arbovirus most monitored is WNV (family Flaviviridae, genus *Flavivirus*). West Nile virus is the leading cause of domestically acquired arboviral disease and has produced the 3 largest arboviral neuroinvasive disease outbreaks ever recorded.^8^ In response to the emergence of WNV in the Western Hemisphere, the US Congress provided funding to establish ArboNET, an electronic surveillance system for arboviral diseases in the United States, and to support surveillance activities in affected states and large cities. Surveillance consists of 2 distinct but complementary measures: (1) a network monitoring WNV activity in mosquito vectors and nonhuman vertebrates and (2) a passive system that records WNV human disease, which can be used to quantify disease burden and identify seasonal, geographic, and demographic patterns of morbidity and mortality in humans.^9^

The principal objective of WNV surveillance is to provide a robust data stream for public health officials and community-based mosquito management programs that quantifies the intensity of virus transmission and can be used as an evidence-based decision support tool.^10,11^ Ideally, this surveillance should also support accurate real-time WNV forecasting. Such a forecasting system would provide public health officials and mosquito control programs with expected likelihoods describing how outbreaks may progress. These forecasts would, in turn, support an evidence-based decision-making approach for targeting control of infected mosquito populations (ie, larviciding [insecticide applications targeting mosquito larvae] and adulticiding [insecticide applications targeting adult mosquitoes]), alerting the public to future periods of elevated WNV spillover transmission risk, and identifying when to intensify blood donor screening. However, a forecasting tool must be tested in real time to determine whether the forecasting apparatus and observational network are sufficient to support accurate operational prediction.

In a recent study,^12,13^ we showed that accurate and reliable forecasts of WNV outbreaks can be made using WNV surveillance data and a mathematical model representing the transmission dynamics of WNV among mosquitoes and birds, as well as spillover to humans. This model system was able to retrospectively forecast mosquito infection rates prior to the week of mosquito peak infection and to forecast accurately the seasonal total number of human WNV cases prior to when the majority of cases were reported. However, for this forecasting system to operate effectively in real time, timely data streams are needed. Mosquito monitoring practices vary around the country and are influenced by local socioeconomic factors, the tax base, and the public and political will to budget for mosquito surveillance.^6,14,15,16^ In addition, the reporting of human WNV disease has long lag times that influence our understanding of how an outbreak is progressing and challenge our ability to generate accurate real-time forecasts.

We report the results from real-time forecasting during the 2017 WNV season in 4 mosquito vector control districts (Chicago, Illinois^17^; Coachella Valley, California^18^; Suffolk County, New York^19^; and St Tammany Parish, Louisiana^20^) (eFigure 1 in the Supplement) as well as operational challenges associated with these efforts. Each week during the 2017 season, observations of human WNV cases and mosquito infection rates were assimilated using a data assimilation method,^17^ and a probabilistic forecast was generated in real time using 2 separate prediction models.^12,13^ We report the accuracy of these real-time forecasts and quantify the association between reporting lags and forecast accuracy.

## Methods

This analysis used 2 forecasting systems, a baseline model with no temperature forcing^13^ and a model that includes environmental forcing (average daily temperature [ie, climatology] for the region) by accounting for temperature modulation of the extrinsic incubation period for mosquitos.^12^ Both models used a standard susceptible-infected-recovered epidemiological construct and were optimized using a data assimilation method and 2 observed data streams: mosquito infection rates and reported human WNV cases. The methodological approach and its application in real-time infectious diseases forecasting are briefly described later in this section. For further details on the validation of the WNV model-inference forecasting systems, see the articles by DeFelice et al.^12,13^ The remainder of the Methods section describes how we used the observed reporting lags from the 2017 WNV season to evaluate the associations of reporting delays with forecast accuracy for 110 historical outbreaks.^12^ We followed the Consolidated Health Economic Evaluation Reporting Standards (CHEERS)^21^ reporting guideline to report the difference in likelihood of an accurate forecast given current reporting lags relative to ideal reporting of all data in real time. This research received institutional review board approval from Columbia University, which waived requirements for participant informed consent because the study used deidentified data.

### Mosquito Data

Abatement districts carried out weekly mosquito surveillance subject to budgetary constraints, the severity of WNV, mosquito nuisance problems, and weather. This surveillance was conducted using gravid, carbon dioxide–baited, light, and sentinel traps, depending on the mosquito abatement district and the species being trapped. The total number of traps set each week within a season and mosquito abatement district varied. For more details on the testing protocol, differences among abatement district collections, mosquito types, and how data were transferred for forecasting, see the eAppendix and eTable in the Supplement.

All mosquito pools reported at the time of forecast were used for model optimization. A maximum likelihood approach was used to estimate the weekly prevalence of infected mosquitoes^22^ given the number of mosquitoes tested in each pool and detection of virus. The prevalence of infected mosquitoes represents both noninfectious mosquitoes, for which the virus is only in the midgut, and infectious mosquitoes, for which the virus has spread to the salivary glands. The eTable in the Supplement presents statistics on mosquito collections by abatement district, including the primary vector, trap type, variability in traps per week, variability in mosquito pools per week, weeks forecasted (weeks with active monitoring after WNV virus was identified), the estimated peak mosquito infection rate (peak magnitude), the week of peak infection rates (peak timing), the number of *Culex* pools assayed for WNV, and the number of WNV-positive *Culex* pools.

### Observed Human Cases

In the United States, WNV is a nationally notifiable disease. Medical professionals are required to report a positive case of WNV to their local health department within 7 days of diagnosis; California requires reporting within 1 day.^23^ Once a case is reported, either of neuroinvasive or nonneuroinvasive (West Nile fever) disease (eAppendix in the Supplement), the local health department opens an investigation and confirms the case. On confirmation, the case is reported to the local mosquito abatement district. At that point, the human case record is available for assimilation into the model and can be backdated using the onset-of-illness date. For details on the differences in human case reporting among districts, as well as reporting lags between onset of illness and reporting to health departments and confirmation of the cases, see the eAppendix in the Supplement.

To better understand human case reporting lags, historical information from the city of Chicago^24^ was obtained describing the lag time between the date of illness onset and the date a suspected WNV case was reported to the local health department. Unfortunately, similar historical information was not available describing the typical period for determining whether a suspected case is probable or confirmed. Only observations from the 4 study locations forecast during the 2017 outbreak were used to estimate the reporting lag from disease onset date to when the investigation of a human WNV case was closed.

### Forecasts

The forecast system relies on 3 components: (1) a core compartmental epidemiological model representing the transmission dynamics of WNV among mosquitoes and birds, as well as spillover to humans^12,13^; (2) WNV surveillance data, ie, vector mosquito WNV infection rates and reported human WNV cases; and (3) a data assimilation method (here, the ensemble adjustment Kalman filter^25^). The data assimilation method uses the surveillance data to recursively inform and optimize an ensemble of model simulations and, in so doing, provide an improved, posterior estimate of the true state as well as estimates of unobserved state variables and parameters. The forecasting is then generated in 2 successive steps. First, an ensemble of model simulations is recursively optimized using the ensemble adjustment Kalman filter and weekly observations of mosquito infection rates and human WNV cases until the week at which a forecast is to be initiated (in real time, this is the current week). Through the recursive ensemble adjustment Kalman filter optimization, model variables and parameters are better aligned with the local dynamics of the outbreak as thus far observed. Next, a forecast is generated by integrating the optimized ensemble of model simulations through to the end of the season. For further details on the WNV model-inference forecasting systems, see the eAppendix in the Supplement or the articles by DeFelice et al.^12,13^

In real time, the future progression of an outbreak is unknown; thus, a reference point for the forecasts is needed. Here, the forecast reference is defined as forecast lead time, which is the number of weeks before or after the predicted peak of infected mosquitoes. This predicted peak is defined as the maximum weekly mosquito infection rate from the mean of the ensemble of simulations. This point can occur either in the posterior (the period for which observations have been assimilated) or in the mean ensemble forecast, depending on the trajectory of the forecast. Forecast lead time was calculated by subtracting the week of predicted peak mosquito infection from the week of forecast generation (in real time, the current week).^26^

Expected accuracies were derived from historical forecast performance for the expected forecast lead time. A forecast was deemed accurate if (1) it peaked within 1 week of the observed peak of infected mosquitoes; (2) the maximum mosquito infection rate was within 25% of the observed peak infection rate; (3) the total number of human cases over the entire season was within 25% or 1 case of the total number of reported cases, whichever was larger; and (4) total infected mosquitoes were within 25% of the observed.

### Association of Observational Lags With Forecasting Accuracy

To better understand the factors associated with forecast accuracy, retrospective forecasts for 110 historical outbreaks^12^ were run with mock reporting delays generated from the 2017 real-time forecasting results (Table). The 110 historical outbreaks were from 12 geographically diverse US counties. Two of the real-time forecast counties (Suffolk County and St Tammany Parish) were part of this analysis. For more details on the 110 retrospective forecast sites and years, see the article by DeFelice et al.^12^

**Table.** Different Reporting Delay Scenarios for West Nile Virus

Scenario	Human Case Lag Distribution, Mean (SD), wk^a^	Mosquito Testing Lag, wk
All data in real time	0	0
All health data in real time, mosquito testing state	0	1
Health department reporting lag, mosquito testing district	2.1 (1.4)	0
Health department confirmation lag, mosquito testing district	5.5 (2.3)	0
Health department reporting lag, mosquito testing state	2.1 (1.4)	1
Health department confirmation lag, mosquito testing state	5.5 (2.3)	1

^a^ Reporting scenarios were generated using a Poisson distribution.

The generated reporting lags represent different reporting scenarios. For mosquito reporting lags, we used a 1-week lag to represent the delay between mosquito trapping and viral testing similar to the reporting lag seen when mosquito abatement districts send samples to the state for viral testing (IM_1_). We compare these lags with mosquito abatement districts that do in-house viral testing and have test results within the same week (IM_0_). To capture the variability in human case reporting, we compared 3 scenarios: (1) all human cases were reported within the week of illness (HC no lag) and all cases are compliant with the national notifiable disease guidelines, (2) human case reporting represented observed lags associated with case reporting to the health department (HC reported), and (3) human case reporting represented the lags associated with health department confirmation (HC confirmed). For these retrospective forecasts, human case reporting lags were randomly selected from a Poisson distribution.

## Results

Real-time forecasts were generated each week during the 2017 season following assimilation of the most recent available information. For the 4 locations, 37 human WNV cases were reported, and 212 179 collected mosquitoes were tested for WNV in 7064 pools (500 pools [7%] were positive). The accuracy of each real-time forecast by calendar week of forecast is shown in eFigure 2 through eFigure 5 in the Supplement. Overall, the real-time forecasts were able to estimate accurately the timing and magnitude of the peak of infected mosquitoes and the total number of infected mosquitoes for the season in real time prior to the peak timing of infected mosquitoes; however, forecasts of human WNV cases showed little skill (the term *skill* is used to describe a model’s measure of prediction accuracy to the observation being predicted). The breakdown in forecasting human cases occurred 1 to 2 weeks prior to the infected mosquito peak, a critical time when spillover events begin. During this period, the forecasts began to underestimate the total number of human WNV cases for the season.

**Figure 1.**
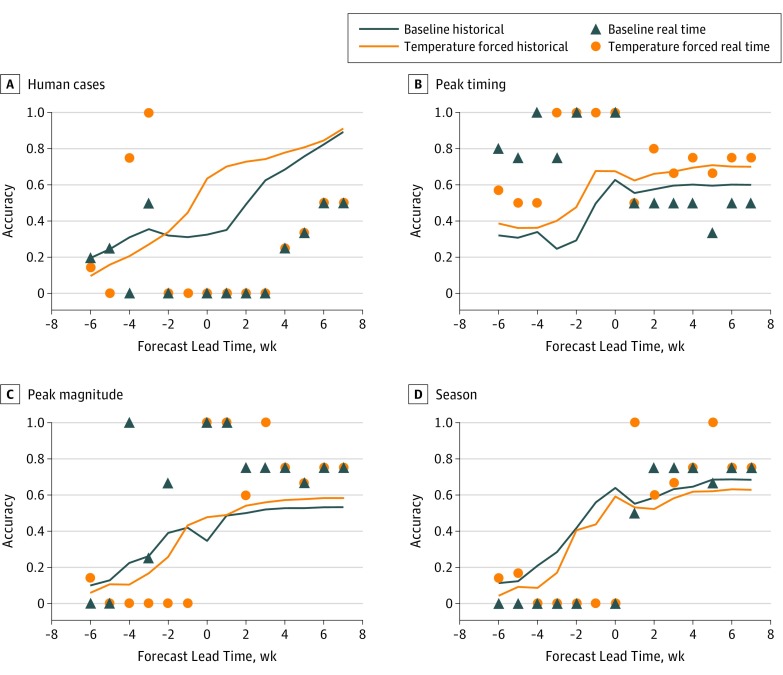
The Fraction of Accurate Real-Time Forecasts The temperature-forced model (orange circle) and baseline model (blue triangle) are reported as a function of lead week for the metrics human West Nile virus cases, peak timing (week of peak mosquito infection rates), peak infection rate, and total infectious mosquitoes. Real-time forecasts are compared against historically accurate forecasts for both the temperature-forced model (orange line) and baseline model (blue line) as a function of lead week for each metric.

**Figure 2.**
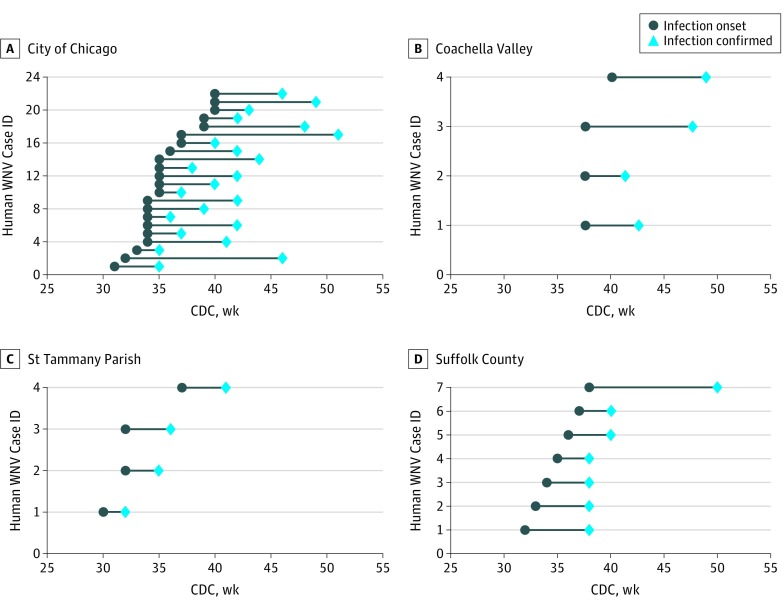
West Nile Virus (WNV) Human Case Observations and Reporting Delays During 2017 at the 4 Study Sites The gray circles represent the week of illness onset and the blue diamonds indicate the week each case was confirmed and reported to the public. The lag time between onset of illness and case confirmation is highly variable, ranging from 2 weeks to 14 weeks. CDC indicates Centers for Disease Control and Prevention; ID, identification number.

Real-time forecast accuracy for the 4 locations was then compared with the accuracy previously derived during retrospective forecast^12,13^ (Figure 1). Forecast accuracy for the peak week of mosquito infection rates was close to that generated for retrospective outbreaks. The temperature-forced forecasts oscillated around the historical probability of accurately forecasting each lead week, whereas the baseline forecasts were biased somewhat below the retrospective forecasts. The real-time peak magnitude forecasts did better than the retrospective predictions at 4 and 0 weeks prior to the predicted peak for the baseline and temperature-forced forecasts, respectively. The seasonal total number of infected mosquitoes was accurately forecast beginning 1 week past the predicted peak. Human case forecasts showed some skill 6 to 3 weeks prior to the predicted peak of infected mosquitoes but were not able to accurately forecast cases from 2 weeks prior to 3 weeks after the predicted peak (lead weeks −2 to 3), a forecast period on which we will now focus. As we will show, some of this forecast error was due to human case reporting lags (Figure 2).

**Figure 3.**
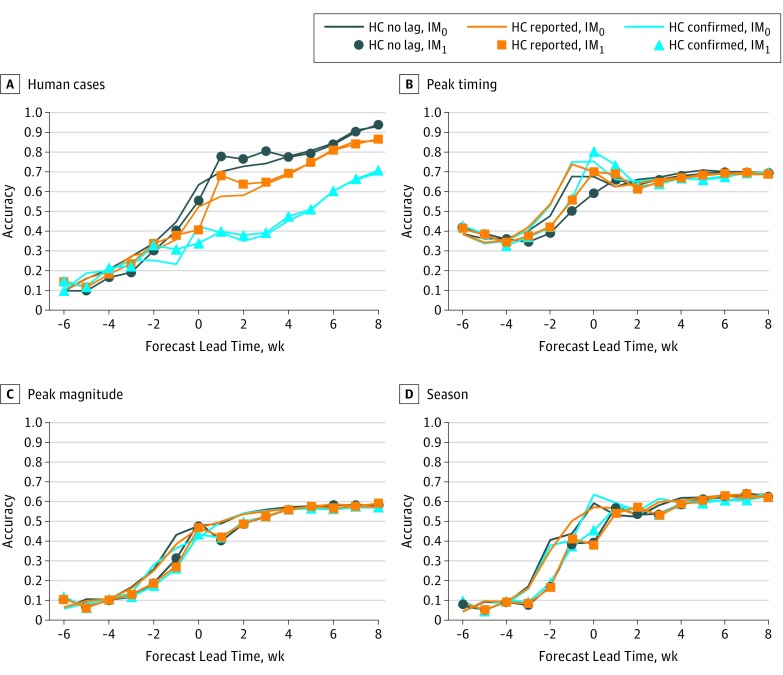
The Temperature-Forced Model Under Different Disease Reporting Scenarios The fraction of accurate retrospective forecasts plotted as a function of lead week for the metrics human West Nile virus cases, peak timing (week of peak mosquito infection rates), peak mosquito infection rate, and total infectious mosquitoes. Each line represents a different reporting scenario. Lines have no infectious mosquito lag (IM_0_), whereas lines with circles, squares, and triangles have a 1-week lag between trapping and reporting of infectious mosquitoes (IM_1_). The navy lines represent forecasts run with no human case reporting lag (HC no lag); orange lines represent the lag between illness onset and reporting to public health departments (HC reported mean [SD], 2.1 [1.4] weeks); and the blue lines represent the lag between onset and confirmation (HC confirmed mean [SD], 5.5 [2.3] weeks).

**Figure 4.**
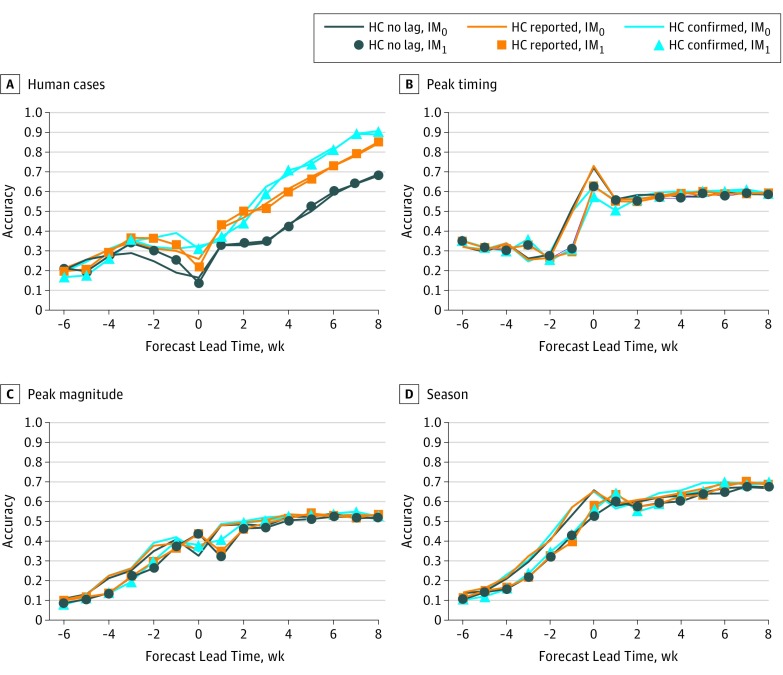
The Baseline Model Under Different Disease Reporting Scenarios The fraction of accurate retrospective forecasts plotted as a function of lead week for the metrics human West Nile virus cases, peak timing (week of peak mosquito infection rates), peak mosquito infection rate, and total infectious mosquitoes. Each line represents a different reporting scenario. Lines have no infectious mosquito lag (IM_0_) whereas lines with circles, squares, and triangles have a 1-week lag between trapping and reporting of infectious mosquitoes (IM_1_). The navy lines represent forecasts run with no human case reporting lag (HC no lag); orange lines represent the lag between onset and reporting to public health departments (HC reported mean [SD], 2.1 [1.4] weeks); and the blue lines represent the lag delay between onset and confirmation (HC confirmed mean [SD], 5.5 [2.3] weeks).

Using 110 historical outbreaks from multiple locations around the country, we evaluated how reporting lags of human cases are associated with forecast accuracy under the scenario of no mosquito testing lag (an onsite laboratory) and different human case reporting lags (Figure 3, Figure 4, and the Table). There was a mean (SD) lag of 2.1 (1.4) weeks (range, 0-8 weeks) between disease onset and when the local health department received the data. This lag reduced average forecast accuracy for total human cases in the temperature-forced and baseline forecasts by 10% and 2%, respectively, for lead weeks −2 to 3, the period when 47% of human cases were reported. Once the health department receives a potential case of WNV, it needs to be confirmed. The combined delay due to notification of the health department and confirmation of the case produced a mean (SD) lag of 5.5 (2.3) weeks (range, 2-14 weeks) between onset of illness and data release to the public during 2017 in the 4 study regions (Figure 2). During lead weeks −2 to 3, this longer combined lag time was associated with reduced average forecast accuracy of 26% and 14% for the temperature-forced and baseline forecasts, respectively.

In addition to accounting for the human case reporting lags, we also evaluated the associations between the reporting lag for mosquito infection rates and processing occurring at a state laboratory. Suffolk County^19^ and St Tammany Parish^20^ used a state laboratory, which subsidized testing but delayed processing. St Tammany Parish and Suffolk County had lags of 4 to 10 days and 5 to 11 days, respectively, between mosquito trapping and state provision of test results. The mosquito reporting lag associated with processing occurring at a state laboratory was a mean (SD) of 6.6 (2.6) days; thus, we represented this lag as a 1-week delay of mosquito infection rate reporting (Figure 3, Figure 4, and the Table). This 1-week lag had minimal association with human case forecasting accuracy; however, it was associated with reduced forecasting accuracy for the 3 mosquito outcomes. For both the baseline and temperature-forced models, forecast accuracy during lead weeks −2 to 3 decreased by approximately 5% for all 3 indicators.

## Discussion

This study highlights some of the current challenges impeding operational implementation of real-time arbovirus infectious disease forecasts in the United States. Specifically, we found that current delays in reporting of confirmed human WNV cases and infectious mosquitoes were associated with decreased real-time forecasting accuracy and utility as an evidence-based decision support tool.

Currently, human case reporting has significant lags related to communication from medical professionals to local health departments, laboratory confirmation, and the interview process used to determine where and when the infection was acquired. In addition to these lags, sharing these data (ie, location and date of disease onset) with local mosquito abatement districts may be limited owing to confidentiality constraints. In the future, accelerating and standardizing the appropriate transfer of timely human case information is needed.

Mosquito lags are shorter and associated with less reduction in accuracy than human case reporting lags but could also be shortened. The mosquito-testing lags were 5 to 8 days longer in state-run laboratories than onsite laboratories. The cost of this mosquito-testing lag was seen prior to the peak of mosquito infection rates, a critical period during which a better understanding of viral prevalence may provide more scientific guidance on decisions directing specific mosquito control practices. Evidence suggests that the timing of 2 key mosquito control interventions, larviciding and adulticiding, is critical for reducing the impact of WNV on humans.^16,27,28^ The costs of switching from state-run to onsite mosquito infection assessment varies. For example, for St Tammany Parish, the cost would be $15 per test for onsite testing compared with $1.50 per test for tests run by the state. In contrast, Suffolk County is fully subsidized by the state and would likely need to budget $15 to $30 per sample for onsite testing. A full assessment of the costs of switching to onsite testing across different abatement districts is beyond the scope of the present study and is left for future work.

For surveillance data and forecasting to help support more targeted and cost-effective interventions, they must have minimal reporting lags, be analyzed quickly, and be presented in easy-to-understand visualizations depicting the current state of circulating virus with probabilities of the likely progression to spillover in a given area. Thus, it is important to develop and standardize a way to communicate risk among mosquito abatement districts, public health departments, and medical professionals to prevent the spread of arbovirus infections. All 3 stakeholders play a unique role in preventing an arbovirus outbreak, and the information each group collects is valuable to the others. Providing medical professionals with a better understanding of entomological risk will give them another data point to help guide clinical differential diagnosis. In return, medical professionals could provide public health departments and mosquito abatement districts with more rapid diagnostic information on spillover events and the current status of an outbreak. High-quality outbreak data reported in a timely fashion are a needed component for forecasting systems, which will help enable better-targeted and more cost-effective interventions.

### Limitations

This study has limitations. In 2017, real-time forecasting of WNV was conducted in 4 unique geographical locations across the United States. Although these sites capture a variety of governing structures between health department and vector control district, they represent only a small fraction of the vector control districts across the United States. Thus, the data used for confirmed human case reporting lags may underrepresent or overrepresent the time it takes to confirm each case. In addition to the time lag between onset and confirmation, human case reporting lags could only be derived from historical outbreaks in the city of Chicago. The city of Chicago reports human WNV cases electronically, which may reduce the reporting lag relative to other health departments. Chicago has also experienced multiple large outbreaks; as a consequence, clinicians may be more aware of reporting practices than in other parts of the country, and reporting lags may underrepresent the rest of the country (even though WNV is a national notifiable disease for which a positive case must be reported to local health department within 7 days of diagnosis). In addition, the study is biased toward mosquito abatement districts and health departments where WNV is endemic and resources are devoted toward surveillance.

## Conclusions

Delays in reporting human WNV cases and infectious mosquito information hinder outbreak surveillance and corrupt real-time forecasting accuracy. During 2017 at 4 studied sites, infectious mosquito reporting lags were short enough that skillful forecasts could still be generated for mosquito indicators; however, human WNV case lags were too great to support accurate forecasting. WNV forecasting is potentially an important evidence-based decision support tool; however, for this tool to support more targeted and cost-effective interventions, more resources are needed to reduce data reporting lags.

## Notes

Supplement. **eAppendix. **Data and Forecasts **eFigure 1.** Four Mosquito Abatement Districts in 4 Different States **eFigure 2.** Forecasts Generated Each Week During the 2017 WNV Outbreak for the City of Chicago **eFigure 3.** Forecasts Generated Each Week During the 2017 WNV Outbreak for Coachella Valley **eFigure 4.** Forecasts Generated Each Week During the 2017 WNV Outbreak for St Tammany Parish **eFigure 5.** Forecasts Generated Each Week During the 2017 WNV Outbreak for Suffolk County **eTable.** Overview of Mosquito Infection Rates and Mosquito Data Used to Generate Real-Time Forecasts During the 2017 WNV Season **eReferences**

## References

[1] Shepard DS, Undurraga EA, Halasa YA, Stanaway JD The global economic burden of dengue: a systematic analysis. Lancet Infect Dis. 2016;16(8):-. doi:10.1016/S1473-3099(16)00146-8 27091092

[2] Nash D, Mostashari F, Fine A, ; 1999 West Nile Outbreak Response Working Group The outbreak of West Nile virus infection in the New York City area in 1999. N Engl J Med. 2001;344(24):1807-1814. doi:10.1056/NEJM200106143442401 11407341

[3] Fischer M, Staples JE; Arboviral Diseases Branch, National Center for Emerging and Zoonotic Infectious Diseases, CDC Notes from the field: chikungunya virus spreads in the Americas—Caribbean and South America, 2013-2014. MMWR Morb Mortal Wkly Rep. 2014;63(22):500-501. 24898168 PMC5779358

[4] Gulland A Zika virus is a global public health emergency, declares WHO. BMJ. 2016;352:i657. doi:10.1136/bmj.i657 26839247

[5] Weaver SC, Barrett AD Transmission cycles, host range, evolution and emergence of arboviral disease. Nat Rev Microbiol. 2004;2(10):789-801. doi:10.1038/nrmicro1006 15378043 PMC7097645

[6] Rosenberg R, Lindsey NP, Fischer M, Vital signs: trends in reported vectorborne disease cases—United States and territories, 2004-2016. MMWR Morb Mortal Wkly Rep. 2018;67(17):496-501. doi:10.15585/mmwr.mm6717e1 29723166 PMC5933869

[7] Keeling MJ, Rohani P Modeling Infectious Diseases in Humans and Animals. Princeton, NJ: Princeton University Press; 2008.

[8] Centers for Disease Control and Prevention ArboNET Database: West Nile virus statistics and maps. https://www.cdc.gov/westnile/statsmaps/index.html. Accessed December 15, 2018.

[9] Centers for Disease Control and Prevention West Nile virus disease cases and deaths reported to CDC by year and clinical presentation, 1999-2014. http://www.cdc.gov/westnile/resources/pdfs/data/1-wnv-disease-cases-by-year_1999-2014_06042015.pdf. Accessed November 3, 2015.

[10] Nasci R, Fischer M, Lindsey N, West Nile Virus in the United States: Guidelines for Surveillance, Prevention, and Control. Fort Collins, CO: Centers for Disease Control and Prevention; 2013.

[11] Nasci RS Monitoring and controlling West Nile Virus: are your prevention practices in place? J Environ Health. 2013;75(8):42-44. 23621056

[12] DeFelice NB, Schneider ZD, Little E, Use of temperature to improve West Nile virus forecasts. PLoS Comput Biol. 2018;14(3):e1006047. doi:10.1371/journal.pcbi.1006047 29522514 PMC5862506

[13] DeFelice NB, Little E, Campbell SR, Shaman J Ensemble forecast of human West Nile virus cases and mosquito infection rates. Nat Commun. 2017;8:14592. doi:10.1038/ncomms14592 28233783 PMC5333106

[14] Healy JM, Reisen WK, Kramer VL, Comparison of the efficiency and cost of West Nile virus surveillance methods in California. Vector Borne Zoonotic Dis. 2015;15(2):147-155. doi:10.1089/vbz.2014.1689 25700046 PMC4340646

[15] National Association of County and City Health Officials Mosquito control capabilities in the U.S. https://www.naccho.org/uploads/downloadable-resources/Mosquito-control-in-the-U.S.-Report.pdf. Accessed October 1, 2017.

[16] Tedesco C, Ruiz M, McLafferty S Mosquito politics: local vector control policies and the spread of West Nile Virus in the Chicago region. Health Place. 2010;16(6):1188-1195. doi:10.1016/j.healthplace.2010.08.003 20709590

[17] West Nile Virus (WNV) Mosquito Test Results 2017 https://data.cityofchicago.org/Health-Human-Services/West-Nile-Virus-WNV-Mosquito-Test-Results/jqe8-8r6s#column-menu. Accessed November 1, 2017.

[18] Henke J, Wittie J. *Mosquito Pooled WNV Test Record 2017* Indio, CA: Coachella Valley Mosquito and Vector Control District; 2017.

[19] Campbell SR, Christopher R *Mosquito Pooled WNV Test Record 2017* Yaphank, NY: Suffolk County Department of Health Services Arthropod-Borne Disease Laboratory; 2017.

[20] Caillouet KA *Mosquito Pooled WNV Test Record 2017* Slidell, LA: St Tammany Parish Mosquito Abatement District; 2017.

[21] Husereau D, Drummond M, Petrou S, Consolidated health economic evaluation reporting standards (CHEERS) statement. Cost Effect Res Allocation. 2013;11(1):6. 10.1186/1478-7547-11-6 PMC3607888 23531194

[22] Biggerstaff BJ Confidence intervals for the difference of two proportions estimated from pooled samples. J Agric Biol Environ Stat. 2008;13(4):478-496. doi:10.1198/108571108X379055

[23] California Department of Public Health Title 17, California Code of Regulations (CCR) §2500, §2593, §2641.5-2643.20, and §2800-2812.

[24] City of Chicago WNV cases 2007-2017 dates. https://data.cityofchicago.org/widgets/jqe8-8r6s. Accessed April 2, 2019.

[25] Anderson JL An ensemble adjustment Kalman filter for data assimilation. Mon Weather Rev. 2001;129(12):2884-2903. doi:10.1175/1520-0493(2001)129<2884:AEAKFF>2.0.CO;2

[26] Shaman J, Karspeck A, Yang W, Tamerius J, Lipsitch M Real-time influenza forecasts during the 2012-2013 season. Nat Commun. 2013;4:2837. doi:10.1038/ncomms3837 24302074 PMC3873365

[27] Petersen LR, Brault AC, Nasci RS West Nile virus: review of the literature. JAMA. 2013;310(3):308-315. doi:10.1001/jama.2013.8042 23860989 PMC4563989

[28] Carney RM, Husted S, Jean C, Glaser C, Kramer V Efficacy of aerial spraying of mosquito adulticide in reducing incidence of West Nile virus, California, 2005. Emerg Infect Dis. 2008;14(5):747-754. doi:10.3201/eid1405.071347 18439356 PMC2600250

