# Supplementary material for: Modeling and Surveillance of Reporting Delays of Mosquitoes and Humans Infected With West Nile Virus and Associations With Accuracy of West Nile Virus Forecasts
Source: JAMA Netw Open. 2019 Apr 26;2(4):e193175. doi: 10.1001/jamanetworkopen.2019.3175 (PMC6487631; doi:10.1001/jamanetworkopen.2019.3175)
Supplement: Supplement. — eAppendix. Data and Forecasts eFigure 1. Four Mosquito Abatement Districts in 4 Different States eFigure 2. Forecasts Generated Each Week During the 2017 WNV Outbreak for the City of Chicago eFigure 3. Forecasts Generated Each Week During the 2017 WNV Outbreak for Coachella Valley eFigure 4. Forecasts Generated Each Week During the 2017 WNV Outbreak for St Tammany Parish eFigure 5. Forecasts Generated Each Week During the 2017 WNV Outbreak for Suffolk County eTable. Overview of Mosquito Infection Rates and Mosquito Data Used to Generate Real-Time Forecasts During the 2017 WNV Season eReferences [file jamanetwopen-2-e193175-s001.pdf]

Supplementary Online Content

DeFelice NB, Birger R, DeFelice N, et al. Modeling and surveillance of reporting delays of mosquitoes and humans infected with West Nile virus and associations with accuracy of West Nile virus forecasts. *JAMA Netw Open*. 2019;2(4):e193175. doi:10.1001/jamanetworkopen.2019.3175

eAppendix. Data and Forecasts
eFigure 1. Four Mosquito Abatement Districts in 4 Different States
eFigure 2. Forecasts Generated Each Week During the 2017 WNV Outbreak for the City of Chicago
eFigure 3. Forecasts Generated Each Week During the 2017 WNV Outbreak for Coachella Valley
eFigure 4. Forecasts Generated Each Week During the 2017 WNV Outbreak for St Tammany Parish
eFigure 5. Forecasts Generated Each Week During the 2017 WNV Outbreak for Suffolk County
eTable. Overview of Mosquito Infection Rates and Mosquito Data Used to Generate Real-Time Forecasts During the 2017 WNV Season
eReferences

This supplementary material has been provided by the authors to give readers additional information about their work.

© 2019 DeFelice NB et al. *JAMA Network Open.*

eAppendix: Data and Forecasts

Data

Mosquito Data

Collected mosquitoes were identified to species level and counted, and a portion of mosquitoes were pooled and assayed for the presence of WNV. These assays were performed using real-time reverse transcription-polymerase chain reaction (rtPCR). The number and size of the pools tested for WNV varied each week. Two of the abatement districts, the city of Chicago[1] and Coachella Valley[2], ran in-house laboratories, whereas Suffolk County[3] and St. Tammany Parish[4] used a state laboratory.

Results from mosquito pool assays were provided to Columbia University. Two of the mosquito abatement districts transferred these data by email (Suffolk County and St. Tammany Parish); data for Coachella valley was uploaded to the California state server[5] and then transferred by email; and the City of Chicago posted its assay results on an online data portal from which these data were downloaded.

Observed Human Cases

There were slight differences in the route of human case reporting among districts. The St. Tammany Parish abatement district received human case information from the Louisiana State health department, and Suffolk County received human case information from the Suffolk County health department. Both then provided these data for use in this study. Riverside County Health Department provided their data directly to our study team when they provided the information to the Coachella Valley mosquito abatement district. The City of Chicago released weekly reports detailing the total number of human WNV cases[6]. Each week these reports were updated with the total number of human cases reported over the season. The City of Chicago health department

© 2019 DeFelice NB et al. *JAMA Network Open.*

then emailed our team the date of illness onset associated with each reported case. Each week as new information related to onset was reported, the observations for past weeks were updated.

Human case reporting lags were highly variable. Lags from disease onset to confirmation were due to reporting delays between either health care professionals and the health department, or the health department work-up to confirm the case of WNV. Cases reported to the health department from medical professionals are considered suspected based on lab and clinical evaluation. The CDC clinical criteria for arboviral disease is broken down into neuroinvasive disease and non-neuroinvasive disease. Neuroinvasive disease includes findings of meningitis, encephalitis, acute flaccid paralysis or other acute signs of neurologic dysfunction not otherwise explained. Non-neuroinvasive disease findings include fever (chills) along with the absence of findings consistent with neuroinvasive disease that are not otherwise explained. These suspected cases are then investigated and considered probable if in addition to the clinical criteria, there are virus-specific IgM antibodies in the serum or CSF (for neuroinvasive disease only). A case of neuroinvasive disease is confirmed if it meets the clinical definition and at least one of the following laboratory criteria:

- Detection of WNV nucleic acid in tissue, blood, CSF, or other body fluid;
- Four-fold or greater change in virus-specific quantitative antibody titer in paired sera;
- Isolation of WNV specific IgM in serum with confirmatory virus-specific neutralizing antibodies in the same or a later specimen; and
- WNV specific IgM antibodies in CSF, with or without a reported pleocytosis.

Non-neuroinvasive disease is defined similarly but in the absence of any findings of arborvirus in the CSF, which would be consistent with neuroinvasive disease. In addition, a negative result for other likely explanations of clinical findings is necessary[7].

© 2019 DeFelice NB et al. *JAMA Network Open.*

Forecasts

For each location, 300 simulations (an ensemble) were used and initialized with initial model state variables and parameters randomly selected from a uniform distribution with defined ranges[8]. The first forecast was generated the week of the first observation of infected mosquitoes after the 21st calendar week. Each subsequent week, new available observations of human WNV cases and mosquito infection rates were assimilated into the model using the EAKF in order to optimize the ensemble of simulations and better represent current local outbreak dynamics. Forecasts were then generated by integrating the ensemble, without further parameter adjustment, to the end of the calendar year. Over the course of the season, this process was repeated weekly, with each successive forecast using an additional week of observations (in real time, the most up-to-date information) for data assimilation. Each 300-member ensemble forecast was repeated 10 times with different randomly selected initial conditions. For more details on the validation of the WNV forecast model-inference system see DeFelice et al.[8,9].

© 2019 DeFelice NB et al. *JAMA Network Open.*

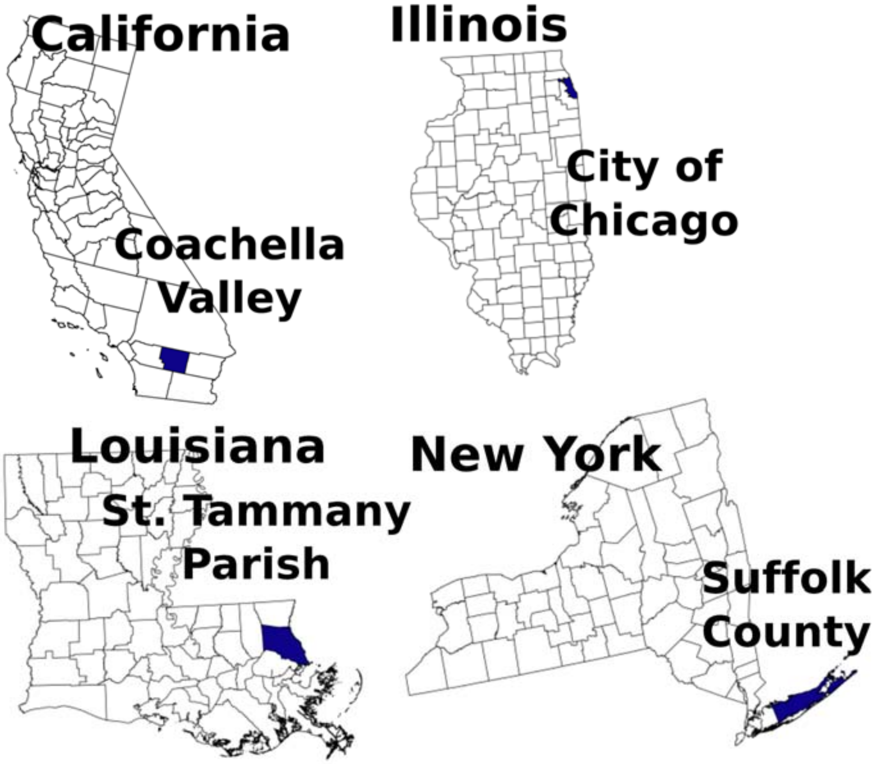

eFigure 1. Four mosquito abatement districts in 4 different states: Coachella Valley, CA, St Tammany Parish, LA, Suffolk County, NY, and the City of Chicago, IL.

© 2019 DeFelice NB et al. *JAMA Network Open.*

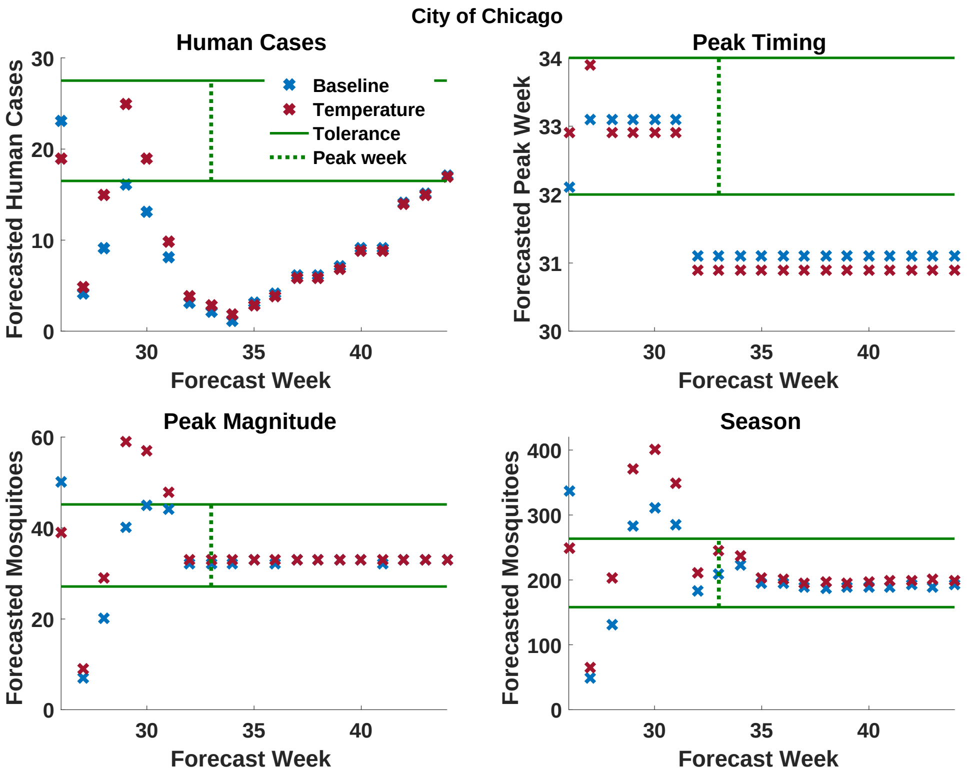

eFigure 2. Forecasts generated each week during the 2017 WNV outbreak for the City of Chicago. Weekly forecasts are shown for both temperature-forced forecasts (red x) and a baseline model forecast (blue x). The horizontal green lines represent the tolerance for an accurate forecast and the vertical dotted green line represents the peak week of infectious mosquito numbers.

© 2019 DeFelice NB et al. *JAMA Network Open.*

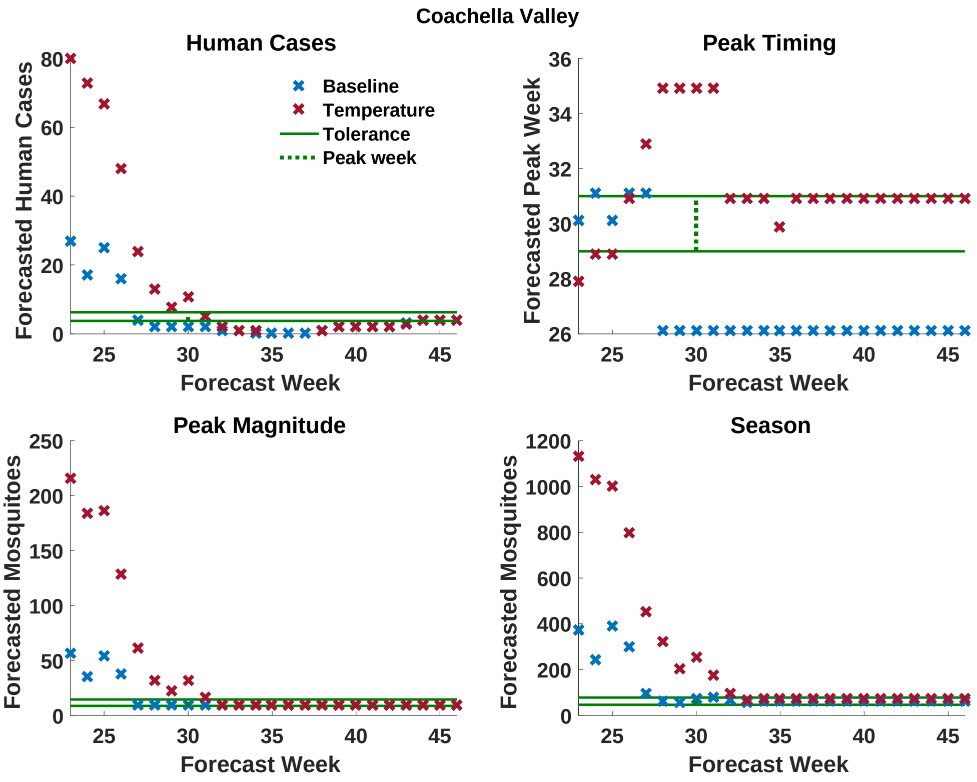

eFigure 3. Forecasts generated each week during the 2017 WNV outbreak for Coachella Valley. Weekly forecasts are shown for both temperature-forced forecasts (red x) and a baseline model forecast (blue x). The horizontal green lines represent the tolerance for an accurate forecast and the vertical dotted green line represents the peak week of infectious mosquito numbers.

© 2019 DeFelice NB et al. *JAMA Network Open.*

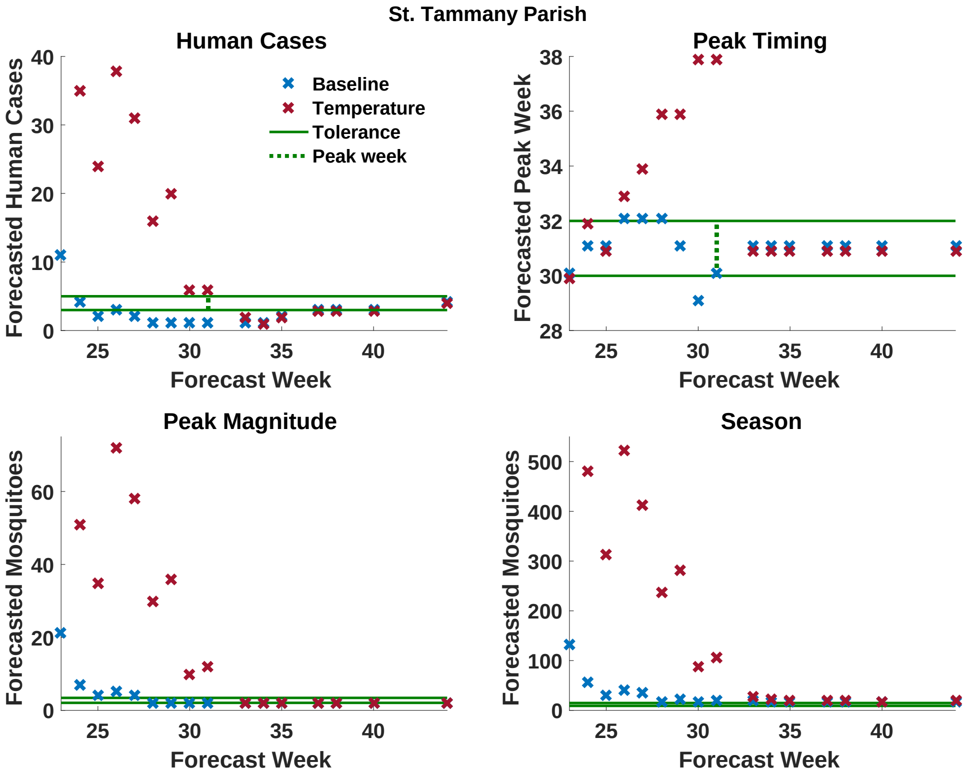

eFigure 4. Forecasts generated each week during the 2017 WNV outbreak for St. Tammany Parish. Weekly forecasts are shown for both temperature-forced forecasts (red x) and a baseline model forecast (blue x). The horizontal green lines represent the tolerance for an accurate forecast and the vertical dotted green line represents the peak week of infectious mosquito numbers.

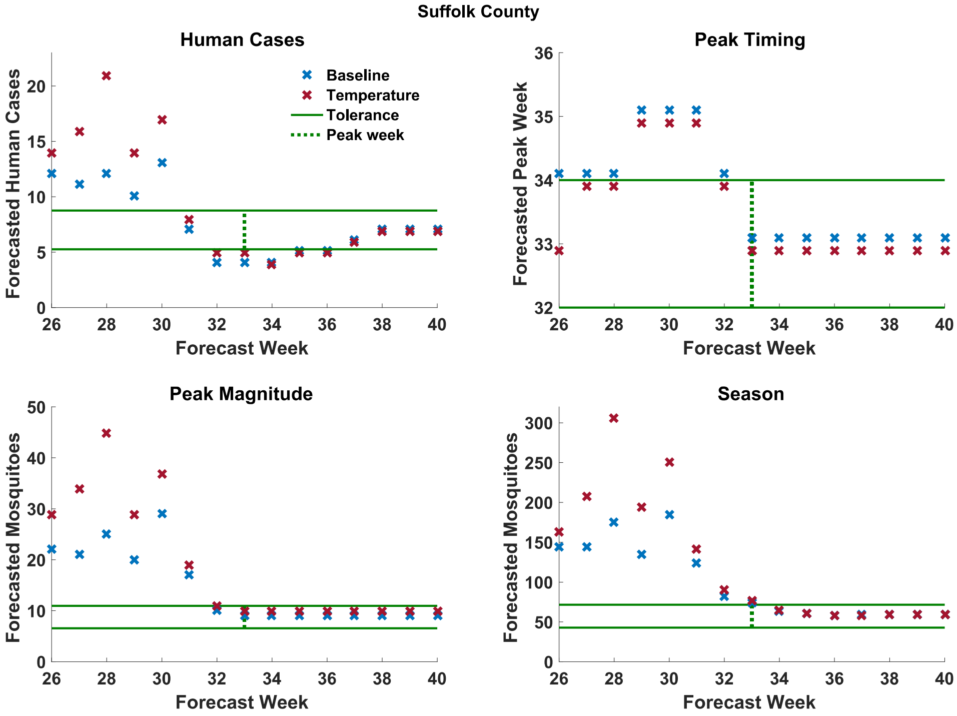

eFigure 5. Forecasts generated each week during the 2017 WNV outbreak for Suffolk County. Weekly forecasts are shown for both temperature-forced forecasts (red x) and a baseline model forecast (blue x). The horizontal green lines represent the tolerance for an accurate forecast and the vertical dotted green line represents the peak week of infectious mosquito numbers.

© 2019 DeFelice NB et al. *JAMA Network Open.*

eTable. **Overview of mosquito infection rates and mosquito data used to generate real-time forecasts during the 2017 WNV season.**

Mosquito abatement district	Primary WNV vector	Trap type	Weeks forecasted	Positive weeks	Peak infection rate* (95% CI)	Peak timing^ (CDC Week)	No. of *Culex* pools tested (mosquitoes)	No. of WNV-positive *Culex* pools	Average number of pools per week (Min, Max)	Average number of traps per week with mosquitoes (Min, Max)	Average infectious mosquito reporting lag (Min, Max)
City of Chicago	*Cx. pipiens* and *Cx. restuans*	Gravid and BG sentinel	25 to 43	15	36.2 (24.3, 52.1)	8/19/2017 (33)	1,499 (14,100)	176	100<br>(51, 180)	53<br>(29, 64)	1.4<br>(1,7)
Coachella Valley	*Cx. quinquefasciatus and Cx. tarsalis*	Gravid, CO2-light, and BG sentinel	23 to 46	27	11.6 (7.4, 17.5)	7/29/2017 (30)	2,592 (80,379)	168	96<br>(35, 165)	58<br>(29,75)	0.4<br>(0,7)
St. Tammany Parish	*Cx. quinquefasciatus*	CO2-light, and Gravid	22 to 44	12	2.7 (1.2, 5.7)	8/5/2017 (31)	1,715 (71,426)	34	62<br>(24, 96)	35<br>(17, 43)	6<br>(4,10)
Suffolk County	*Cx. pipiens* and *Cx. restuans*	Gravid and CO2-light	26 to 40	14	8.8 (5.3, 13.9)	8/19/2017 (33)	1,258 (46,274)	122	70<br>(24, 90)	38<br>(14,47)	9.2<br>(5, 11)

*Per 1,000 mosquitoes estimated using the Maximum Likelihood Estimation
^ Last day of the week

© 2019 DeFelice NB et al. *JAMA Network Open.*

eReferences:

1. West Nile Virus (WNV) Mosquito Test Results. 2017. https://data.cityofchicago.org/Health-Human-Services/West-Nile-Virus-WNV-Mosquito-Test-Results/jqe8-8r6s - column-menu. Accessed November 1, 2017.
2. Henke J, Wittie J. Mosquito Pooled WNV Test Record 2017. Coachella Valley Mosquito and Vector Control District 2017.
3. Campbell SR, Christopher R. Mosquito Pooled WNV Test Record 2017. Suffolk County Department of Health Services Arthropod-Borne Disease Laboratory 2017.
4. Caillouet KA. Mosquito Pooled WNV Test Record 2017. St. Tammany Parish Mosquito Abatement District 2017.
5. Barker C, Kramer V, Reisen W. Decision support system for mosquito and arbovirus control in California. *Earthzine: An IEEE Publication.* 2010.
6. City of Chicago. West Nile Virus Surveillance Reports. 2017; https://www.cityofchicago.org/city/en/depts/cdph/supp_info/infectious/west_nile_virus_surveillancereports2011.html. Accessed November 1, 2017.
7. Centers for Disease Control and Prevention. Arboviral Diseases, Neuroinvasive and Non-neuroinvasive 2015 Case Definition 2017; https://wwwn.cdc.gov/nndss/conditions/west-nile-virus-disease/case-definition/2015/, 2017.
8. DeFelice NB, Little E, Campbell SR, Shaman J. Ensemble forecast of human West Nile virus cases and mosquito infection rates. *Nature Communications.* 2017;8:14592.
9. DeFelice NB, Schneider Z. , Little E., Barker C., Caillouet K. A., Campbell S. R., Damian D., Irwin P., Jones H. M.P., Townsend J. , and Shaman J. . Use of temperature to improve West Nile virus forecasts. *Plos Computational Biology*. 2018;14(3).

© 2019 DeFelice NB et al. *JAMA Network Open.*
